# Hydrogen Peroxide and Nitric Oxide are Involved in Salicylic Acid-Induced Salvianolic Acid B Production in *Salvia miltiorrhiza* Cell Cultures

**DOI:** 10.3390/molecules19055913

**Published:** 2014-05-08

**Authors:** Hongbo Guo, Xiaolin Dang, Juane Dong

**Affiliations:** State Key Laboratory of Crop Stress Biology for Arid Areas, College of Life Sciences, Northwest A&F University, Yangling 712100, China; E-Mails: hbguo@nwsuaf.edu.cn (H.G.); dongj@nwsuaf.edu.cn (X.D.)

**Keywords:** *Salvia miltiorrhiza*, salicylic acid, hydrogen peroxide, nitric oxide, salvianolic acid B, cell culture

## Abstract

Hydrogen peroxide (H_2_O_2_) and nitric oxide (NO) are key signaling molecules in cells whose levels are increased in response to various stimuli and are involved in plant secondary metabolite synthesis. In this paper, the roles of H_2_O_2_ and NO on salvianolic acid B (Sal B) production in salicylic acid (SA)-induced *Salvia miltiorrhiza* cell cultures were investigated. The results showed that H_2_O_2_ could be significantly elicited by SA, even though IMD (an inhibitor of NADPH oxidase) or DMTU (a quencher of H_2_O_2_) were employed to inhibit or quench intracellular H_2_O_2_. These elicited H_2_O_2_ levels significantly increased NO production by 1.6- and 1.46 fold in IMD+SA and DMTU+SA treatments, respectively, and induced 4.58- and 4.85-fold Sal B accumulation, respectively. NO was also markedly elicited by SA, in which L-NNA (an inhibitor of NO synthase) and cPTIO (a quencher of NO) were used to inhibit or quench NO within cells, and the induced NO could significantly enhance H_2_O_2_ production by 1.92- and 1.37-fold in L-NNA+SA and cPTIO+SA treatments, respectively, and 3.27- and 1.50-fold for Sal B accumulation, respectively. These results indicate that elicitation of SA for either H_2_O_2_ or NO was independent, and the elicited H_2_O_2_ or NO could act independently or synergistically to induce Sal B accumulation in SA-elicited cells.

## 1. Introduction

Plants provide a wide variety of secondary metabolites useful to mankind, such as pharmaceuticals, food additives, flavors, and other industrial materials. Often high-value secondary metabolites are found in low abundance in Nature [1]. Because of the high cost and unreliability of harvesting products from natural resources to fulfill the needs of pharmaceutical industry, strategies such as treating with elicitors to increase their production have been used in plant cell cultures [2,3]. Many secondary metabolites have been observed in much higher concentrations in elicited cultured cells than in whole plants of the same species [1,2].

Salicylic acid (SA), a phenolic compound broadly distributed in plants, has been recognized as a regulatory signal mediating plant response to abiotic stresses such as drought, chilling, heavy metal tolerance, heat and osmotic stress [1]. Most of research on this hormone focuses on its role in local and systemic response against microbial pathogens, and on defining the transduction pathway leading to gene expression induced by SA [4]. However, knowledge about its role in enhancing the production of secondary metabolites as an elicitor is rather limited.

Hydrogen peroxide (H_2_O_2_) and nitric oxide (NO) are key signaling molecules produced in cells in response to various stimuli and involved in a diverse range of plant signal transduction processes [5]. One of the earliest responses of plants to various stimuli is the production of active oxygen species (AOS) such as H_2_O_2_, and superoxide anion. It has been found that the H_2_O_2_-scavenging enzyme catalase has been isolated as a SA-binding protein, which can be inactivated by SA and its active analogs [6], but treatment with SA can increase H_2_O_2_ levels, and thus H_2_O_2_ is proposed to be the downstream signal of SA [7]. However, this fact cannot harmonized with the observation that SA is required for induction of systemic acquired resistance (SAR) proteins by H_2_O_2_, and that catalase inactivation has deleterious effects on plant health [8]. In fact, the relation between H_2_O_2_ and SA in both activation of defense against pathogens and accumulation of secondary metabolites is currently not clear.

In plants, NO has been identified as a second messenger during hypersensitive response (HR), and can enhance plant growth, delay senescence and activate phytoalexin production. Some studies have highlighted the putative links between NO and oxidative stress, which suggest that NO plays a prominent role during HR, and complex functions that are both complementary and competitive to those of H_2_O_2_ [9,10].

Although considerable advances have been made in understanding the role of redox signals during both HR and SAR, a critical aspect requiring further investigation is how signals such as H_2_O_2_ and NO can be specific [7]. The related problem is how plants respond to these signals when using SA-induced signaling system. The complex interactions between H_2_O_2_, NO and SA may evolve from the need for pathogen defense pathways and production of secondary metabolites.

Salvianolic acid B (Sal B) is one of effective compounds listed in quality control of Danshen, the root and rhizome of *Salvia miltiorrhiza* Bunge, which is used as a traditional Chinese herbal drug for removing blood stasis, alleviating pain, promoting the circulation of blood, promoting menstruation, tranquilizing the brain, and treating cardiovascular and cerebrovascular disease [11,12]. Phenolic compounds have been proven to have significant bioactivities such as antioxidant, anti-ischemia reperfusion, and antithrombotic effects, and Sal B, one of them, shows great free radical scavenging and antioxidant activity [13,14].

The aim of this work is to reveal the role of H_2_O_2_ and NO in SA-induced Sal B production in *S*. *miltiorrhiza* cell cultures. For this purpose, both dimethylthiourea (DMTU) and carboxy-2-phenyl-4,4,5,5-tetramethylimidazoline-1-oxyl-3- oxide (cPTIO) were employed as scavengers of H_2_O_2_ and NO, respectively, and imidazole (IMD) was used to inhibit the enzyme activity of NADPH oxidase.

## 2. Results and Discussion

### 2.1. H_2_O_2_ Production in SA-Induced Cells and Its Effect on Sal B Accumulation

**Figure 1:**
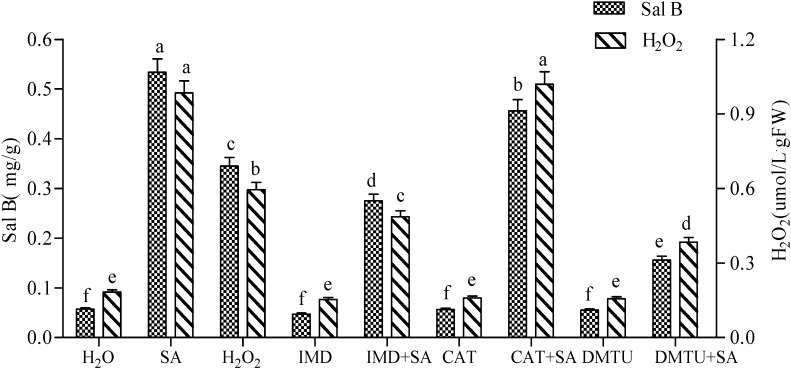
Effects of salicylic acid (SA), H_2_O_2_, imidazole (IMD), catalase (CAT), dimethylthiourea (DMTU) and complex treatments on the production of Sal B and H_2_O_2_ in *Salvia miltiorrhiza* cell cultures. The concentrations for SA, H_2_O_2_, IMD, CAT and DMTU were 22 mg·L^−^^1^, 10 mmol·L^−^^1^, 100 μmol·L^−^^1^, 100 U and 500 μmol·L^−^^1^, respectively. SA was added to subcultured *Salvia miltiorrhiza* cells after 8 d culture, and both contents of H_2_O_2_ and Sal B were determined in the subsequent 8 h and 2 d, respectively, with three replications. Different lowercase letters represented significance at 0.05.

Different treatments using SA, IMD, CAT and DMTU were carried out in order to investigate the production of H_2_O_2_ in SA-induced cells and its effect on Sal B production. The contents of H_2_O_2_ and Sal B in SA-induced *Salvia miltiorrhiza* cell cultures were 4.38- and 8.37-fold higher than that of H_2_O, respectively (Figure 1). By contrast, the exogenous H_2_O_2_ also significantly increased the contents of H_2_O_2 _and Sal B (3.25- and 6.05-fold, respectively), but both were lower than those of SA-induced cells.

IMD is an inhibitor of NADPH oxidase in the plasma membrane, which can inhibit the production of H_2_O_2_ by inactivating NPDPH oxidase. DMTU is a H_2_O_2_ quencher that can effectively remove H_2_O_2_ within cells. Neither IMD nor DMTU treatment affected the contents of Sal B and H_2_O_2_ when compared with H_2_O, indicating both chemicals with such concentrations have no deleterious effect on cell growth and their ability to produce H_2_O_2_ and Sal B. Although both IMD+SA and DMTU+SA treatments significantly decreased the contents of H_2_O_2_ and Sal B, when they were compared with SA-elicited cells, both contents were significantly higher than that of H_2_O. This indicates that H_2_O_2_ can still be markedly produced in SA-elicited cells, even though both IMD and DMTU are added to scavenge or quench intracellular H_2_O_2._ At the same time, this H_2_O_2_ elicited by SA significantly increases the accumulation of Sal B (Figure 1).

CAT is a scavenger of H_2_O_2_, which cannot pass through the cell membrane, and thus the exogenous CAT cannot scavenge intracellular H_2_O_2_. In this paper, the contents of Sal B and H_2_O_2_ in CAT treatment were roughly equivalent to H_2_O (Figure 1), indicating CAT at such a concentration has no harmful effect on cells. Similarly, there was no difference between CAT+SA treatment and SA-elicited cells in terms of H_2_O_2_ production, which may correlate with the fact that the exogenous CAT cannot scavenge H_2_O_2_ within cells. The Sal B accumulation in CAT+SA treatment, meanwhile, was significantly lower than that of SA-elicited cells, which may be due to the fact that CAT can bind some of the SA and thus diminish its elicitation effect on Sal B production.

**Figure 2:**
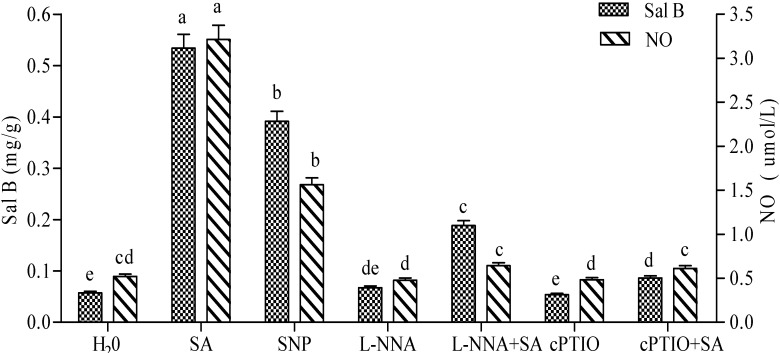
Effects of SA, sodium nitroprusside (SNP), N^ω^-nitro-L-arginine (L-NNA), carboxy-2-phenyl-4,4,5,5-tetramethylimidazoline-1-oxyl-3-oxide (cPTIO) and complex treatments on the production of Sal B and NO in *Salvia miltiorrhiza* cell cultures. The concentrations for SA, SNP, L-NNA and cPTIO were 22 mg L^−1^, 0.5 mmol L^−1^, 2 mmol L^−1^ and 50 μmol L^−1^, respectively. SA was added to subcultured *Salvia miltiorrhiza* cells after 8-d culture, and in the subsequent 4 h and 2 d, both contents of NO and Sal B were determined, respectively, with three replications. Different lowercase letters represented significance at 0.05.

### 2.2. NO Production in SA-Induced Cells and Its Effect on Sal B Accumulation

To investigate the production of NO in SA-induced cells and its effect on Sal B accumulation, treatments including SA, SNP (NO donor), L-NNA and cPTIO were employed. In SA-elicited cells, the contents of NO and Sal B were increased by 5.16- and 8.29-fold, respectively, compared with H_2_O. In comparison, the exogenous SNP increased both contents by 2.99- and 6.80-fold, which were lower than in both SA-elicited cells (Figure 2).

L-NNA is an inhibitor of NO synthase (NOS), which can effectively inhibit the production of NO by inactivating NOS, and cPTIO is a quencher of NO, which can effectively scavenge NO within cells. The results showed that no difference in either Sal B and NO contents was found between L-NNA treatment and H_2_O, or between cPTIO treatment and H_2_O, which indicates that neither L-NNA or cPTIO treatment causes harmful effects on NO production and Sal B accumulation during cell growth. When compared with SA-elicited cells, neither combined L-NNA+SA or cPTIO+SA treatment significantly decreased the contents of Sal B and NO, but both contents were markedly higher than H_2_O with any of the combined treatments, showing that NO still can be efficiently produced in SA-induced cells despite the fact that L-NNA and cPTIO are added to inhibit or quench intracellular NO, and the NO elicited by SA induces a large amount of Sal B accumulation.

### 2.3. Production of NO Elicited by SA and Its Effect on the Accumulation of H_2_O_2_ and Sal B

In order to discover the complex interactions between H_2_O_2_, NO and SA, the production of NO elicited by SA and its effect on the accumulation of H_2_O_2_ and Sal B were investigated by using SNP, L-NNA and cPTIO treatments.

**Figure 3:**
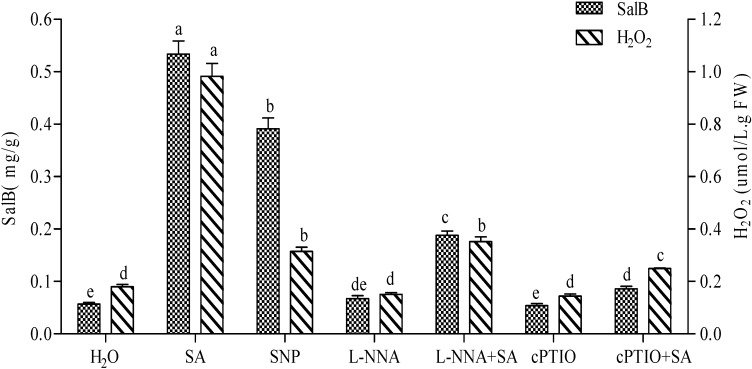
Effects of SA, SNP, L-NNA, cPTIO and complex treatments on the production of Sal B and H_2_O_2_ in *Salvia miltiorrhiza* cell cultures. The concentrations for SA, SNP, L-NNA and cPTIO were the same as Figure 2. The detection time for determining both contents of H_2_O_2_ and Sal B was the same as Figure 2. Different lowercase letters represented significance at 0.05.

On one hand, the contents of H_2_O_2_ and Sal B were slightly decreased in both L-NNA and cPTIO treatments, when compared with H_2_O, which shows that the newly produced NO within cells in either L-NNA or cPTIO treatment (Figure 2) cannot produce more H_2_O_2_ and Sal B than H_2_O (Figure 3). On the other hand, as mentioned above, NO could be significantly elicited by SA in either L-NNA+SA or cPTIO+SA treatment (Figure 1), and H_2_O_2_ production was also increased 1.92- and 1.37-fold, respectively, and Sal B content was enhanced 3.27- and 1.50-fold, respectively (Figure 3). These results indicate that NO elicited by SA significantly generates H_2_O_2_ and thus markedly promotes the accumulation of Sal B.

### 2.4. Production of H_2_O_2_ Elicited by SA and Its Effect on the Accumulation of NO and Sal B

**Figure 4:**
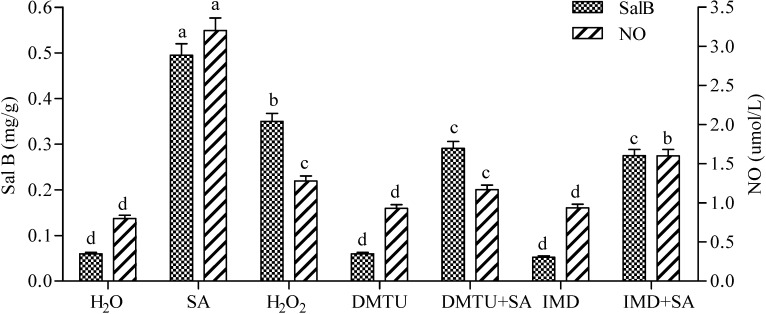
Effects of SA, exogenous H_2_O_2_, IMD, DMTU and complex treatments on the production of Sal B and NO in *Salvia miltiorrhiza* cell cultures. The concentrations for SA, H_2_O_2_, IMD, CAT and DMTU were the same as Figure 1. The detection time for determining both contents of NO and Sal B was the same as Figure 3. Different lowercase letters represented significance at 0.05.

The production of H_2_O_2_ elicited by SA on the accumulation of NO and Sal B were also investigated by using IMD and DMTU in order to illustrate the relationships between H_2_O_2_, NO and SA. Firstly, the contents of NO and Sal B in both IMD and DMTU treatments were slightly increased, but not significantly, showing that the newly produced H_2_O_2_ in IMD and DMTU treatments (Figure 1) is not able to elicit more NO and Sal B than H_2_O (Figure 4). Secondly, as exhibited in Figure 2, H_2_O_2_ could be significantly induced by SA in both IMD+SA and DMTU+SA treatments, and these elicited H_2_O_2_ markedly increased NO production by 2.00- and 1.46-fold, respectively, and enhanced Sal B to 4.58- and 4.85-fold, respectively (Figure 4). These results show that H_2_O_2_ elicited by SA significantly produces NO and thereby stimulating the accumulation of Sal B.

### 2.5. Both H_2_O_2_ and NO Mediate the SA-Elicited Sal B Production

**Figure 5:**
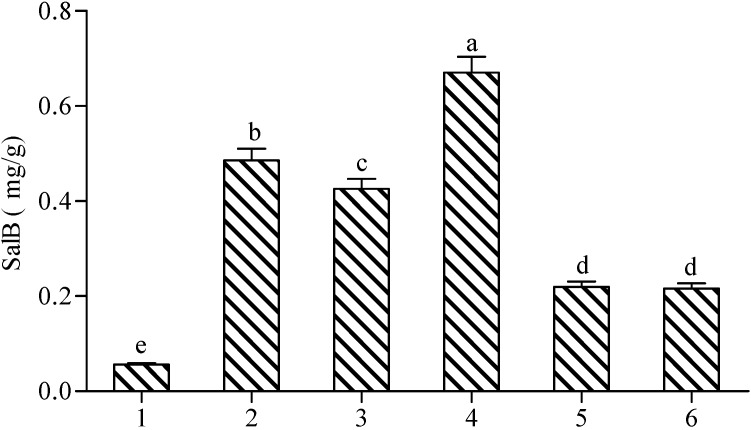
Effects of different treatments on the production of Sal B in *Salvia miltiorrhiza* cell cultures. 1: control (H_2_O); 2: SA; 3: SNP + H_2_O_2_; 4: SNP + H_2_O_2_ + SA; 5: SNP + H_2_O_2_ + DMYU + IMD; 6: H_2_O_2_ + SNP + cPTIO + L-NNA. The detection time for determining Sal B production was the same as Figure 1. Different lowercase letters represented significance at 0.05.

As exhibited in Figure 1 and Figure 2, both H_2_O_2_ and NO could be elicited in SA-induced cells, and each of them could promote the accumulation of Sal B independently. The newly produced NO elicited by SA could induce H_2_O_2_ production and *vice versa*, and the accumulation of Sal B were all significantly enhanced in both situations (Figure 3 and Figure 4). In Figure 5, the content of Sal B was significantly higher after SNP + H_2_O_2_ + DMTU + IMD treatment than that in H_2_O, which also shows that NO can act independently, and so can H_2_O_2_, because the Sal B produced from SNP+H_2_O_2_+L-NNA+cPTIO treatment was significantly higher than that of H_2_O (Figure 5).

On the other hand, the Sal B content in either SNP + H_2_O_2_ + DMTU + IMD or SNP + H_2_O_2_ + L-NNA + cPTIO treatment was significantly lower than that of SNP + H_2_O_2_, which indicates that NO and H_2_O_2_ can act synergistically. This synergistic action was also exhibited between SA and SA+SNP+H_2_O_2_ treatments, in which Sal B production in the former was significantly lower than that of the latter (Figure 5).

### 2.6. Discussion

**Figure 6:**
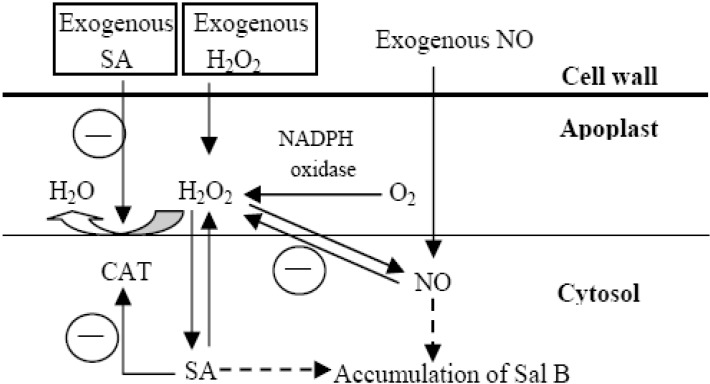
A proposed schematic illustration of H_2_O_2_ and NO roles in SA induced *Salvia miltiorrhiza* cell cultures. The circled “—” shows negative regulation. SA: salicylic acid; CAT: catalase; NO: nitric oxide; Sal B: salvianolic acid B.

SA has been identified as a key component of the SAR signal transduction pathway [15] and can induce gene expression related to biosynthesis and production of secondary metabolites in plants [2,16]. H_2_O_2_ comes to light as a second messenger involved in SA-elicited pathway [8,17]. In this paper, accumulation of H_2_O_2_ and Sal B increases 8.37- and 4.38-fold, respectively, in SA-elicited *S*. *miltiorrhiza* cell cultures (Figure 1 and Figure 2), which is consistent with the report that SA can increase H_2_O_2_ levels [6]. At the same time, exogenous H_2_O_2_ can also enhance both enzyme activities and Sal B production [2], and a 6.05-fold increase of Sal B production was found in this paper. In fact, there are many reports highlighting the fact that H_2_O_2_ mediates the elicitor-induced accumulation of secondary metabolites [2]. On the other hand, if H_2_O_2_ production was inhibited by IMD or quenched by DMTU, the content of H_2_O_2_ did not decrease significantly, which may be attributed to a proposed mechanism of production (Figure 6). In this production mechanism, Ca^2+^, NO, activation of transcriptional factors (TFs) and *TF* genes, SA and ROS signaling may all be involved to produce a certain amount of H_2_O_2_ for the basal level of cell needs [2]. This interplay between SA and ROS signaling also occurs in tobacco and *Arabidopsis*, in which application of H_2_O_2_ and SA induces each other [18,19,20]. Notably, all inhibition caused by IMD, CAT and DMTU can be reversed in SA-elicited cells, of which inactivation of CAT is the most prominent (Figure 1). Researches have revealed that CAT and other H_2_O_2_-scavenging enzymes can be bound and inactivated by SA and its active analogs [6,10]. This may be the most important reason why H_2_O_2_ still can be largely produced in SA-elicited cells, even though CAT has been added (Figure 1 and Figure 6).

In plants, NO has been reported as a second messenger during HR, exhibiting functions that are both complementary and competitive to those of H_2_O_2_ [9,21]. It can also reversibly inhibits cytochrome *c* oxidase and O_2_ uptake in both animal and plant mitochondria [18], and inhibition of cytochrome *c* oxidase activity may increase the electron flow from ubiquinone towards oxygen, thereby stimulating superoxide and H_2_O_2_ formation [10]. This is consistent with our observation that SNP treatment (NO donor) increased H_2_O_2_ by 1.75-fold (Figure 3). On the other hand, NO was found to induce expression of *PAL* and chalcone synthase (*CHS*) [22], and to enhance Sal B production that was 6.86-fold higher in SNP-treated cells than H_2_O (Figure 3). Transcriptional profiling also showed that NO treatment induced some stress- and disease-related signal transduction component genes and defense genes, implying that the NO signal pathway(s) may be related to secondary metabolism [23]. Together, NO may trigger gene expression via at least two pathways, one leads to phenylpropanoid and flavonoid biosynthesis through activating both *TF* and secondary metabolism genes, and a second that leads to NO signaling pathway, including NOS, cGMP, cADP ribose, Ca^2+^ influx, protein kinase and activation of TFs (Figure 6) [2].

If NO was inhibited by L-NNA (NOS inhibitor) or removed by cPTIO (NO scavenger), the contents of NO within cells decreased slightly but not significantly when compared with H_2_O (Figure 2). This phenomenon indicates that there is a mechanism for NO production, in which NR maybe plays an important role involved in H_2_O_2_- and/or ABA-mediated NO production [5,24,25,26]. It has been found that NO levels in the root of wild-type *Arabidopsis* plants have been shown to increase 8-fold after H_2_O_2_ treatment [27]. In this paper, an increase of 60% for NO production was observed after that exogenous H_2_O_2_ was added to cells. Interestingly, NO production would not be inhibited but elevated when IMD and DMTU were added to inhibit the activity of NADPH oxidase and to scavenge H_2_O_2_, respectively (Figure 4). As proposed in Figure 6, when H_2_O_2_ production was inhibited, NO accumulation would be enhanced to release H_2_O_2_ by activating NOS and regulating O_2_ uptake [28], which mainly depends on the second NO signaling pathway mentioned above. The other optional route may be related to SA and ROS signaling, but this needs to depend on the NO signaling pathway to produce NO too.

## 3. Experimental Section

### 3.1. Cell Culture and Treatment

The detailed protocols of *S*. *miltiorrhiza* cell culture and SA elicitation treatment were provided by Dong *et al.* [13]. Stock solutions of H_2_O_2_, DMTU (H_2_O_2_ scavenger, Sigma, Shanghai, China), CAT (Sigma), IMD (NADPH scavenger, Sigma), sodium nitroprusside (SNP, NO donor, Sigma), N^ω^-nitro-L-arginine (L-NNA, inhibitor of NO synthase, Sigma) and cPTIO (NO scavenger, Sigma) were prepared in distilled water and then sterilized after filtration through 0.22 μm membrane. To scavenge H_2_O_2_ and NO in *S*. *miltiorrhiza* cell culture, final concentrations of 500 μmol·L^−^^1^ DMTU and 50 μmol·L^−1^ cPTIO were added separately to the cell culture 30 min before SA treatment. All experiments were performed with three replicates.

### 3.2. Determination of H_2_O_2_ and NO

The calli (0.2–0.3 g) were homogenized with acetone (5 mL) precooled below 4 °C and then ground to a homogenate on ice. The mixture was centrifuged at 10,000 rpm for 10 min at 4 °C, and 5% titanous sulfate (0.5 mL) and concentrated ammonia water (2 mL) were added to 2 mL of supernatant. After mixing, it was centrifuged at 10,000 rpm for 10 min at 4 °C and the supernatant was removed. The pellet was dissolved by 2 mol·L^−^^1^ sulphuric acid (5 mL) after removing pigments by using acetone. This solution was used for H_2_O_2_ determination and the absorbance was measured at 415 nm. The detection was determined after 8 h SA-induction and this point of time was determined by experiment ahead of detection.

The content of NO was determined according to the production of nitrite with Greiss reagent that could oxidize NO to nitrite under acidic conditions [29]. The *S*. *miltiorrhiza* cell culture was leached with 0.22 μm membrane and Greiss reagent (1 mL) was added to 1 mL of filtrate. The mixture was incubated for 30 min at room temperature to convert nitrite into a purple azo-dye and the absorbance was determined at 550 nm. The detection was determined after 4 h SA-induction and this point of time was also determined by experiment ahead of detection.

### 3.3. Sal B Extraction and HPLC Analysis

The *S*. *miltiorrhiza* cells were collected from cell cultures by centrifugation at 1,200 rpm, and then dried at 47.5 °C in an oven to a constant weight. The dried cells (0.05 g) were extracted ultrasonically with methanol-water solution (2 mL, 7:3 v/v) for 45 min. The extract was filtered through a 0.22 μm membrane and the filtrate was obtained for detection.

The content of Sal B was quantified by HPLC analysis that was performed using a Shimadzu (Kyoto, Japan) system equipped with a UV/visible absorbance detector (DAD). A Shim-pack VP-ODS column (250 mm × 4.6 mm, 5 μm) was used at a column temperature of 30 °C. The flow rate was 1 mL·min^−1^ and the injection volume was 10 μL. The DAD detection wavelength was 281 nm. The extraction yield of Sal B was calculated based on the integration of the chromatographic peak areas. More details were described in Dong *et al.* [30].

## 4. Conclusions

The present work shows SA is an effective elicitor inducing accumulation of Sal B in *S*. *miltiorrhiza* cell cultures. H_2_O_2_ could be significantly elicited in SA-induced cells, even though H_2_O_2_ was inhibited or quenched. These elicited H_2_O_2_ significantly increased NO production and Sal B accumulation. NO was also markedly elicited by SA under the circumstances where NO was inhibited or quenched, and the induced NO could significantly enhance H_2_O_2_ production and Sal B accumulation. The elicitation of SA for either H_2_O_2_ or NO was independent, and the elicited H_2_O_2_ or NO could act independently or synergistically to induce Sal B accumulation in SA-elicited cells.
